# Identification of gliadin-binding peptides by phage display

**DOI:** 10.1186/1472-6750-11-16

**Published:** 2011-02-17

**Authors:** Tingsu Chen, Karolina Hoffmann, Sofia Östman, Ann-Sofie Sandberg, Olof Olsson

**Affiliations:** 1 Department of Cell and Molecular Biology, University of Gothenburg, SE-40530, Gothenburg, Sweden; 2 Department of Chemical and Biological Engineering/Food Science, Chalmers University of Technology, SE-41296, Gothenburg, Sweden; 3 Department of Clinical Bacteriology, University of Gothenburg, SE-40530, Gothenburg, Sweden; 4 Department of Plant and Environmental Sciences, University of Gothenburg, SE-40530, Gothenburg, Sweden; 5 Microbiology Institute, Guangxi Academy of Agricultural Sciences, Nanning, Guangxi 530007, PR China

## Abstract

**Background:**

Coeliac disease (CD) is a common and complex disorder of the small intestine caused by intolerance to wheat gluten and related edible cereals like barley and rye. Peptides originating from incomplete gliadin digestion activate the lamina propria infiltrating T cells to release proinflammatory cytokines, which in turn cause profound tissue remodelling of the small intestinal wall. There is no cure for CD except refraining from consuming gluten-containing products.

**Results:**

Phage from a random oligomer display library were enriched by repeated pannings against immobilised gliadin proteins. Phage from the final panning round were plated, individual plaques picked, incubated with host bacteria, amplified to a population size of 10^11 ^to 10^12 ^and purified. DNA was isolated from 1000 purified phage populations and the region covering the 36 bp oligonucleotide insert from which the displayed peptides were translated, was sequenced. Altogether more than 150 different peptide-encoding sequences were identified, many of which were repeatedly isolated under various experimental conditions. Amplified phage populations, each expressing a single peptide, were tested first in pools and then one by one for their ability to inhibit binding of human anti-gliadin antibodies in ELISA assays. These experiments showed that several of the different peptide-expressing phage tested inhibited the interaction between gliadin and anti-gliadin antibodies. Finally, four different peptide-encoding sequences were selected for further analysis, and the corresponding 12-mer peptides were synthesised *in vitro*. By ELISA assays it was demonstrated that several of the peptides inhibited the interaction between gliadin molecules and serum anti-gliadin antibodies. Moreover, ELISA competition experiments as well as dot-blot and western blot revealed that the different peptides interacted with different molecular sites of gliadin.

**Conclusions:**

We believe that several of the isolated and characterised gliadin-binding peptides described here could provide valuable tools for researchers in the field of CD by facilitating studies on localisation and uptake of various gliadin peptides in the small intestine. In future work, the potential of these peptides to detoxify gluten will be investigated.

## Background

Coeliac disease (CD) is a common and complex inflammatory disorder of the small intestine that affects genetically susceptible individuals carrying HLA-DQ2 or -DQ8 haplotypes. Symptoms develop after ingestion of gluten storage proteins (prolamins) from wheat (gliadins), barley (hordeins), rye (secalins), and their crossbred varieties [1,2]. CD can be diagnosed at any age. It can either be asymptomatic or present with a broad spectrum of clinical manifestations. The classical (typical) form of CD is usually characterized by gastrointestinal symptoms like flatulence, vomiting, constipation or persistent diarrhoea, general failure to thrive, mineral and vitamin deficiencies, and weight loss due to malabsorption. Atypical forms, on the other hand, present predominantly with extra-intestinal manifestations that include a blistering skin disease (Dermatitis herpetiformis), iron-deficiency anaemia, osteoporosis, fatigue and neurological complaints [3-6]. The prevalence of CD is estimated to be about 1% in the Western populations [7,8]. Moreover, in recent years the total disease prevalence has increased. The reason for the observed raise is currently unknown and cannot be explained by the increase of CD diagnosis that occurred after introduction of antibody screening [9,10].

In CD patients, peptides that originate from incomplete digestion of gluten prolamins, either in their native form or deamidated by tissue transglutaminase (tTG), bind to HLA-DQ2 or -DQ8 receptors of antigen presenting cells that activate the lamina propria infiltrating CD4^+ ^T cells. As a response the CD4^+ ^T cells release pro-inflammatory cytokines, in particular γ-interferon. Ultimately, this leads to profound tissue remodelling characterised by the atrophy of the small intestinal villi and hyperplasia of crypts [2,11-14]. Active CD is also characterised by high levels of antibodies against tTG and gliadin in the patients' sera. The role of anti-tTG IgA class antibodies is still unclear. However, it has been proposed that they may be involved in the development of mucosal damage [15]. Also IgG class anti-gliadin antibodies have been shown to contribute to the pathogenesis by activating the complement system or inducing antibody-mediated cytotoxicity [16].

T cell epitopes in wheat gluten proteins have been characterised within both gliadins and glutenins. A hierarchy exists within these epitopes. The majority of CD patient-derived intestinal T cell clones recognise α-gliadins, and less frequently γ-gliadins and glutenins [17-20]. The most prominent peptide is a 33-mer of α-gliadins (residues 57-89) that contains six T-cell epitopes. Another fragments, also found in α-gliadins (residues 31-43 and 44-55), seem to be important for the activation of the innate immunity system [18,21-23]. In a recent study gluten-specific T cells from peripheral blood of CD patients challenged either with wheat, barley, rye or a combination of the three cereals were used to identify the immunostimulatory sequences in these grains [24]. The α-gliadin 33-mer was found immunogenic only after the wheat challenge while sequences from ω-gliadin (wheat) and C-hordein (barley) were found to be immunodominant despite the grain consumed.

Currently there is no cure for CD. The only existing therapy is a life-long adherence to a gluten-free (GF) diet [3]. However, several strategies that may in the future serve as alternatives to the GF diet have been proposed. T cell activation may be inhibited by molecules that block peptide binding to HLA-DQ2. Alternatively, inhibition of tissue transglutaminase may prevent gluten deamidation [25]. Supplementation with prolyl endopeptidases (PEPs), enzymes derived from moulds and bacterial strains, or with a mixture of PEP and cysteine endoprotease from germinating barley, which aid in digestion of immunostimulatory gluten peptides into harmless molecules, is under investigation [26-29]. Another possible therapeutic alternative that is currently pursued is a vaccine that contains a mixture of immunodominant peptides that trigger the immune response and are supposed to retrain the immune system of HLA-DQ2 positive CD subjects to tolerate gluten [24,30]. Furthermore, an inhibitor of paracellular permeability (AT-1001, larazotide acetate) has been shown to reduce the intestinal barrier dysfunction, production of pro-inflammatory cytokines, and GI symptoms in CD individuals after gluten exposure [31,32]. Moreover, attempts have been made to degrade toxic gluten sequences during sourdough fermentation with selected lactobacilli strains [33]. Finally, the potential of a linear polymeric binder P(HEMA-*co*-SS) to neutralise gliadin *in vitro *and *in vivo *in mice models have been described [34]. Another alternative could be blocking gliadin domains with synthetic peptides and thus preventing tTG modification and formation of immunostimulatory epitopes.

In the present study we have selected *in vitro *gliadin-binding peptides with the help of phage display. Phage display refers to a molecular method where gene libraries are constructed in filamentous bacteriophage in a way that each individual phage in the population will display a unique peptide or protein on its surface [35]. From such a population, phage that interact with in principle any *a priori *chosen molecule can be isolated and amplified. The concept has been used in many different variations to select and produce novel peptides that bind to target molecules of interest [36-38]. The aim of this work was to identify phage that express peptides specifically binding to different gliadin domains and to identify and characterise the gliadin-binding properties of the chosen individual peptides.

## Results

### Purification and immobilisation of gliadin proteins

**Figure 1:**
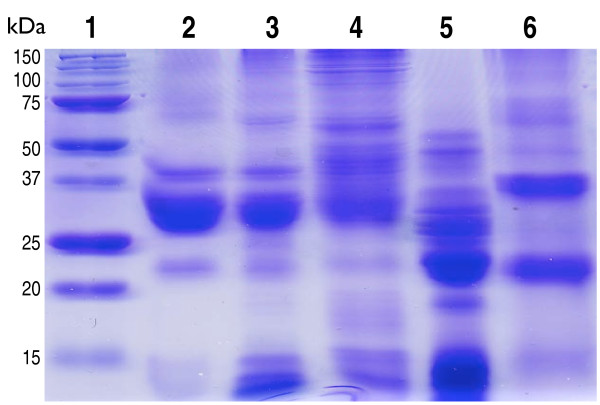
**SDS-PAGE separation of gliadin and gliadin-like proteins**. Lane 1: molecular weight standard; lane 2: gliadin purified from gluten (Sigma) in 40% ethanol; lane 3: gliadin purified from *Triticum aestivum *cv. Surco in 40% ethanol; lane 4: gliadin purified from *Triticum aestivum *cv. Surco in 2 M urea; lane 5: gliadin-like (avenins) proteins purified from *Avena sativa *cv. Leon in 40% ethanol; lane 6: avenins purified from *Avena sativa *cv. Leon in 2 M urea.

**Figure 2:**
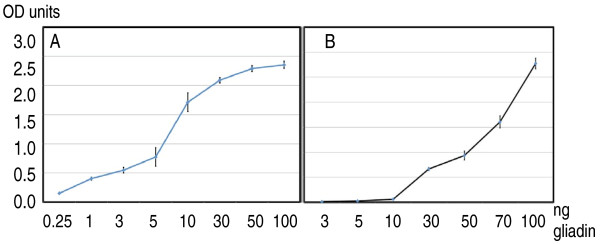
**Quantification of immobilised semi-purified gliadin (Sigma)**. Microtiter wells were coated with increasing amounts of gliadin (ng) as indicated on X-axis. Commercial anti-gliadin antibodies bound to the coated gliadin were quantified by adding a secondary AP-tagged goat anti-human IgG antibody and measured as absorbance at 405 nm (Y-axis). A. Gliadin dissolved in 100 μl 0.1 M NaHCO_3 _(pH 8.6). B. Gliadin dissolved in 100 μl 2 M urea. Error bars indicate variation between two different experiments

Gliadin was semi-purified from commercially obtained gluten (Sigma) and from whole wheat grains as a comparison. Several proteins of varying molecular weights were obtained from both sources (Figure 1). Using a commercial gliadin ELISA kit it was confirmed that the included anti-gliadin antibodies reacted with the semi-purified gliadin fractions. As a control, gliadin-like proteins (avenins) were extracted from oat grains but the commercial antibodies did not, as expected, react with the oat proteins (data not shown). The gliadin preparation was finally dissolved in either 0.1 M NaHCO_3 _or in 2 M urea, added to microtiter plate wells and allowed to bind to plastic. After washing, bound gliadin molecules were quantified with an ELISA assay using the commercial human anti-gliadin IgG antibodies. This confirmed that gliadins indeed had been immobilised to the wells and that the antibodies used recognised both the gliadin NaHCO_3_- and urea-treated molecules. However, the detection limit was reduced about 5 times when gliadins were dissolved in urea indicating that the 3D structure of the antibody-binding sites of gliadin were affected by the buffer used (Figure 2).

### Selection of peptides specifically binding to gliadin peptides by biopanning

Two different phage display libraries, one displaying 12-mer and one displaying 7-mer random peptides, were panned to the immobilised gliadin. In each panning round unbound phage were removed by washing. Remaining bound phage were eluted and allowed to infect *E. coli *cells. After single plaque amplification and phage purification a new round of panning was performed with the obtained phage population. Panning was done both with gliadin dissolved in urea and in NaHCO_3. _Since electrostatic binding of the phage to the target protein weakens with increasing ionic strength, which in turn influences the specificity of the interaction [39], different buffers with different ionic strengths were tested prior the actual panning experiments. After optimising parameters like binding, washing, and elution conditions, a protocol was developed in which the phage recovery increased after each round of panning. Typically five rounds of panning were done. After the final plating, 100 plaques were randomly picked and amplified separately. Phage DNA was then isolated from each isolate, the oligonucleotide inserts sequenced and the deduced amino acid sequence of the displayed peptide determined.

In total, inserted individual oligomer sequences from approximately 1000 phage, selected under a number of different panning conditions, were obtained. Although identical sequences were frequently picked up in independent experiments, altogether more than 160 unique sequences encoding peptides with potential gliadin binding activities were identified (data not shown). All obtained sequences originated from the 12-mer library. Many of the peptides could be crudely divided into subgroups based on sequence similarities. However, more than a half of the peptides showed no obvious sequence similarities to each other. This indicates that the peptide-targeted surfaces in the used gliadin preparations are much diversified.

As a control, panning against microtiter plates coated with BSA was performed. Peptides identified in this way were denoted *control peptides *(CP). Altogether, five different CP sequences were identified. There were no sequence similarities between these control sequences and any of the gliadin binding sequences.

### Rescuing selected phage clones

**Figure 3:**
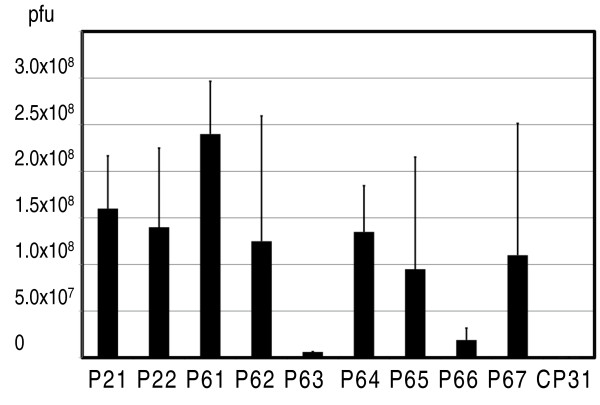
**Phage recovery experiment**. Different phage (1 × 10^11 ^pfu), as indicated on the X-axis, were incubated in gliadin (10 ng) coated microtiter wells. After washing, bound phage were eluted and counted. The Y-axis shows the number of recovered phage given as plaque forming units (pfu). The experiment was replicated three times. Error bars indicate the variation between the experiments.

In order to confirm that the selected phage clones interacted with gliadin proteins, nine different phage populations that had repeatedly been picked up in different panning experiments were chosen. Together, these nine sequences represent 89% of all identified gliadin-binding sequences (Table 1). The remaining approximately 150 sequences thus were found in only 11% of the cases. From each of the nine populations 1 × 10^11 ^pfu were incubated with the gliadin-coated microtiter wells. In addition, a phage population representing a non gliadin-binding control sequence (CP31) was incubated with the gliadin-coated microtiter wells. After extensive washing, remaining phage were eluted and counted. This showed that phage carrying the CP31 control peptide were very poor binders, as only 1 × 10^4 ^pfu were rescued from this population under the conditions used. From the phage that carry specific peptides, on the other hand more than 1 × 10^8 ^pfu were rescued (Figure 3). Phage P61, which was the was most frequently picked up in the panning experiments, displayed the highest relative binding affinity, while phage carrying peptides P21, P22, P62, P64, P65 and P67 showed intermediate affinities (Figure 3).

**Table 1:** Most frequently identified phages and peptides in this study

Peptide	Sequence	Frequency (%)
P61	W H W R N P D F W Y L K	22.5
P64	W H W T W L S E Y P P P	21.5
P22	L E T S K L P P P A F L	12.5
P62	W H W S Q W L S G S P P	8.5
P63	W H R T P Q F W A F P W	7
P21	S V S V G M K P S P R P	5
P66	W H K T P W F W P T N L	5
P67	W H W S W Q P Q R H S P	4
P65	W H W Q Y T P W W R G S	3
**Sum**		**89**
CP31 (control)	A Y Y P Q N H K S N A E	NA

### Selected phage clones hinder anti-gliadin antibody binding to gliadin

**Figure 4:**
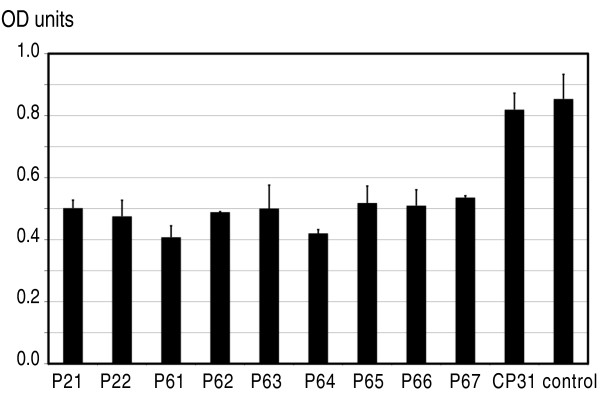
**Gliadin-anti-gliadin antibody interaction in the presence of peptide-displaying phage**. The Y-axis shows different phage displaying peptides tested. Phage (10^11 ^pfu/ml) were added to microtiter wells coated with 10 ng gliadin. After incubation and extensive washing, pooled sera from patients with suspected CD were added. The amount of bound antibody was determined by adding a secondary AP-tagged goat anti-human IgG antibody, and measured as absorbance at 405 nm (Y-axis).

To investigate if the selected, gliadin-interacting phage could hinder antibody binding to gliadin, 1 × 10^11 ^pfu of the same nine phage populations as in the previous experiment (Figure 3) were added to wells coated with 10 ng gliadin. The CP31 phage population as well as a buffer solution without phage were used as controls. To investigate if the peptides not only interfered with commercial anti-gliadin antibody binding but also interfered with the interaction between gliadin and antibodies present in serum from individuals with suspected CD, after extensive washing, pooled sera from 20 individuals was added. Again, the amount of bound antibody was quantified by adding an AP-labelled secondary anti-human IgG antibody. This showed that the greatest signals i.e. most antibodies bound were from the controls where no gliadin-blocking peptides were present (Figure 4). On the other hand, all the selected phage populations blocked the signal to various extents, indicating that the peptides displayed on the phage interfered with the anti-gliadin antibody-gliadin interaction. Wells incubated with phage populations P61 and P64 gave the lowest signal indicating, in agreement with the previous experiment (Figure 3), that these were the most efficient gliadin binders.

### Gliadin binding of synthetic peptides

**Figure 5:**
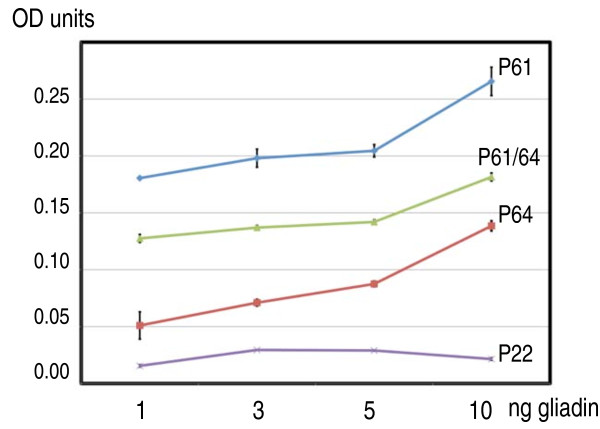
**Binding of synthetic peptides to gliadin proteins**. Microtiter wells were coated with increasing amounts of gliadin (ng) as indicated on the X-axis. Afterwards 10^15 ^molecules (1.67 nmoles) of biotinylated peptides, P61, P64, or P22 were added to the wells. Bound peptides were quantified as absorbance units at 405 nm (OD units) by means of the biotin adduct as described in Methods. P61/64 is a 1:1 mixture of peptides P61 and P64.

Since all selections were based on phage-displayed peptides, we investigated whether synthetic peptides, with the same sequence as those in the selected phage, maintained the gliadin-binding ability also when removed from the steric context of the phage surface. Peptides based on the most frequently identified sequences, P64, P61 and P22 (Table 1) as well as the non-specifically binding control peptide CP31, were synthesised. A biotin label was added at the N-terminus of all the peptides to facilitate detection. To test peptide-gliadin binding, different concentrations of gliadin proteins (110 ng) were immobilised in microtiter wells and 10^15 ^molecules (1.67 nmoles) of the synthetic peptides were added to the wells. After incubation and extensive washing to remove unbound peptides the biotin signals from the remaining peptides were quantified. The results showed that P61 had the highest binding activity, P64 somewhat weaker, and P22 the weakest binding activity (Figure 5), corroborating the experiments with the phage carrying peptides (Figure 3 and 4). No activity at all could be detected from the incubations with the control peptide. As expected, the number of bound peptides increased with increasing gliadin concentrations (Figure 5).

### Gliadin-blocking activity of selected synthetic peptides

**Figure 6:**
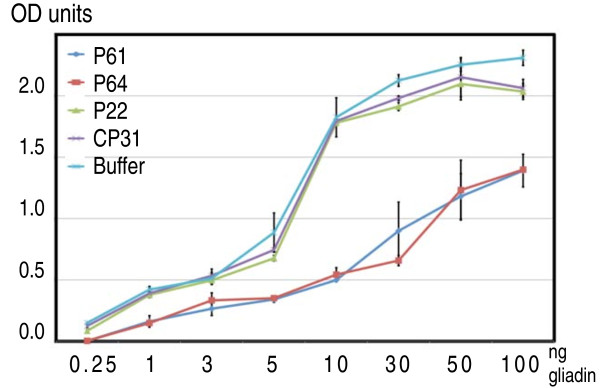
**Inhibition of gliadin/anti-gliadin antibody binding by selected synthetic peptides**. Increasing amounts of gliadin in ng (as indicated on the X-axis) were incubated with 1.67 nmoles of biotin-labelled synthetic peptides (10^15 ^molecules) for 1 h at room temperature. Buffer (0.1 M NaHCO_3_) without peptides was used as a control. The mixes were then added to anti-gliadin monoclonal antibody-coated wells provided with the Immunotech gliadin ELISA kit. After incubation for 1 h at room temperature, gliadin-antibody complexes immobilised in the wells were quantified as described (see Methods) and given as absorbance units at 450 nm (Y-axis). Error bars show the variation in three different experiments.

To confirm that the free peptides could block anti-gliadin antibody-gliadin interactions with similar efficiency as the phage-displayed peptide, 1.67 nmoles (10^15 ^molecules) of the synthetic peptides were mixed with increasing concentrations of gliadin (0.25-100 ng), followed by an incubation of the peptide/gliadin mixture in anti-gliadin antibody coated wells. After incubation and washing away unbound gliadin and peptides, a secondary antibody binding to the solid phase antibody-antigen complex was added, and the amount of complex quantified by means of the tag on the secondary antibody. This showed that the P64 and P61 peptides interfered with or blocked antigenic sites on the gliadin molecules, since fewer signals were obtained with these peptides than with P22, CP31 and the *no peptide *control (Figure 6). Furthermore, the blocking effect was visible in the whole concentration range (0.5-100 ng) of gliadin tested (Figure 6).

**Figure 7:**
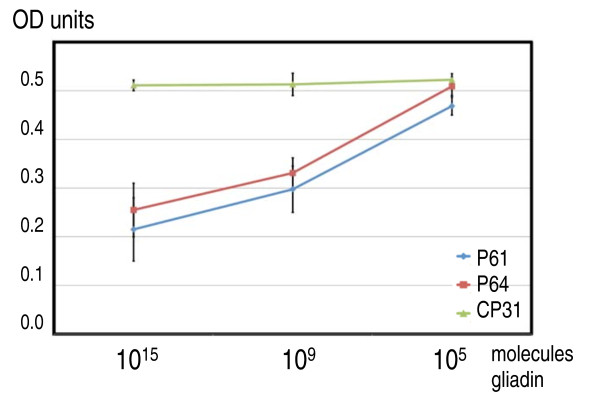
**Concentration dependency of peptide gliadin/anti-gliadin antibody binding inhibition**. Gliadin was diluted to 1 ng/ml and mixed with 1.67 nmoles, 0.167 pmoles, and 0.0167 fmoles (10^15^, 10^9^, and 10^5 ^molecules respectively) of peptides P61, P64, and CP31. Afterwards the gliadin/peptides mixes were added to anti-gliadin, monoclonal antibody-coated wells and incubated for 1 h. The formed gliadin/antibody complexes immobilised in the wells were quantified as described (see Methods) and given as absorbance units at 450 nm (Y-axis).

To further test the binding efficiencies of the peptides, two best peptides P61 and P64 and the control peptide CP31 were diluted in several steps and incubated with 1 ng of gliadin followed the incubation in the antibody-coated microtiter wells. This showed that as little as 10^9 ^peptide molecules (0.167 pmoles) could interfere with the anti-gliadin antibody-gliadin interactions to a level detectable in the experiment (Figure 7).

### Dot blot and western blot analysis

**Figure 8:**
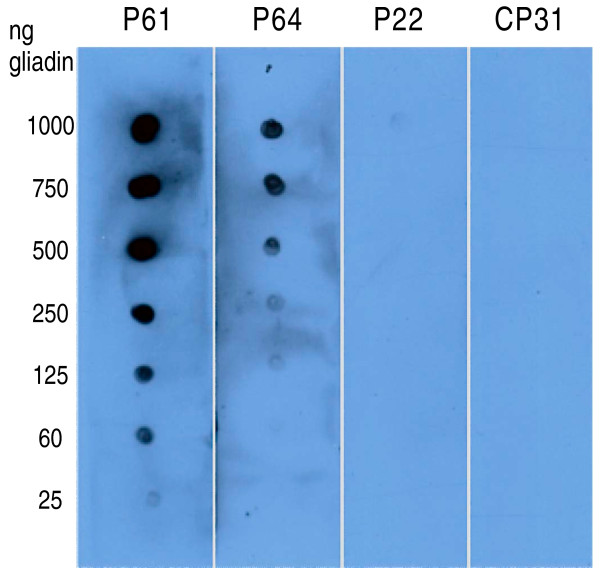
**Dot blot analysis of peptide-gliadin binding**. Different amounts of gliadin proteins, as indicated on the Y-axis, were spotted onto nitrocellulose strips. Each strip was incubated with 1.67 nmoles (10^15 ^molecules) of the peptide indicated on the top of the figure. After washing the biotin signal from the bound peptide was developed as described.

To further verify the physical interaction of the peptides and gliadin, and to elucidate if the peptides preferentially bind to specific proteins in the semi-purified gliadin fraction used here, dot blot and western blot experiments were performed. In the dot blot experiments, increasing concentrations (25 ng/μg) of the gliadin preparation were spotted on filters. The filters were then incubated with either of the P22, P61, P64 or CP31 peptides, and subsequently washed. Bound peptides were quantified with an anti-biotin AP-labelled antibody recognising the biotin tag on the peptides. The results from these analyses again confirmed that the peptides physically interacted with gliadin and as previously, the P61 peptide had the highest binding activity, followed by the P64 peptide. No binding could be detected with the P22 and the CP31 peptides (Figure 8).

**Figure 9:**
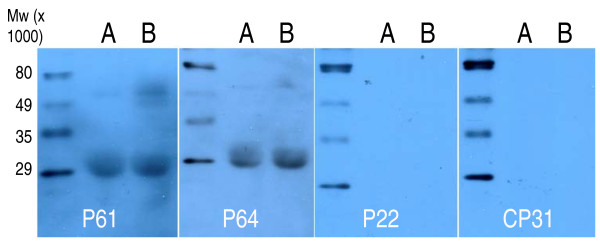
**Western blot analysis of peptide-gliadin binding**. 10 μg of gliadin extracted from Sigma gluten (A) or 10 μg of gliadin extracted from Surco wheat (B) was loaded on a polyacrylamide gel. Separated proteins were transferred to a nitrocellulose membrane. Each membrane was incubated with 1.67 nmoles (10^15 ^molecules) of the peptide indicated at the bottom of the figure. After washing the biotin signal from the bound peptide was developed as described.

In the western blot experiments, gliadin proteins were separated on SDS-PAGE gels and blotted to nitrocellulose membranes. By using the P61, P64, P22 or CP31 peptides as probes and again detecting peptides bound to the filter by means of the secondary AP-labelled anti-biotin antibody it became clear that, as in the dot blot experiments, P61 and P64 showed the strongest binding (Figure 9). Both these peptides interacted with proteins in the 29-30 kDa range, and in addition P61 also bound to proteins between 49-70 kDa. Thus, the two peptides have overlapping but distinct binding specificities. A similar pattern was obtained both when analysing the gliadin extracted from Sigma gluten and gliadin extracted from wheat (Figure 9).

## Discussion

Wheat gluten consists of a complex mixture of proteins that based on their common structures can be divided into three groups: a high molecular weight (HMW) group that contains HMW-glutenin subunits with Mr ~67-88 kDa; a medium molecular weight (MMW) group containing ω-gliadin proteins with Mr ~34-55 kDa; and a low molecular weight (LMW) group with α/β-, γ-gliadins and LMW-glutenin subunits with Mr ~28-39 kDa [40-43]. Several of the glutenin and gliadin proteins contain repeated proline and glutamine residues, especially QQPFP and PQQPF motifs, which are resistant to complete digestion by gastric and pancreatic enzymes [18]. The repeats can trigger immune response and appear to be especially important for the specific gluten peptide recognition by CD4^+ ^T cells [17,44]. Previously, it was shown that phage display might be a useful technology to identify peptides that bind to gliadin residues, although no sequences were shown [38]. Here, we extended this work to include several different gliadin proteins to increase the probability of identifying phage that bound to reactive surfaces (Figure 1). Glutamine residues within gliadin can be deamidated by tissue transglutaminase (tTG), which will further enhance the pathologic immune response [45]. If blocking peptides efficiently inhibit recognition of gliadin by tTG they will most probably aid in limiting the development of T cell epitopes. In a recently published study we have shown that when in complexes with the selected blocking peptides, the *in vitro *enzymatic modification of gliadin by tTG was reduced by ~one-third [46].

There is no cure for CD. The only available therapy is a life-long exclusion of gluten from a diet. The variety of bakery and pastry gluten-free products is limited and the price is higher than their gluten containing equivalents [47]. Moreover, these products often do not meet the dietary requirements, as they tend to be high in fat and low in fibre as compared to gluten-containing equivalents [47,48]. Furthermore, naturally gluten-free grains and flours can be contaminated with gluten during fieldwork, transport, processing or in a store if grains are kept in open containers [49,50]. One alternative strategy would be to neutralise minor contaminations in e.g. oat, rice or maize products by mixing in molecules that block or digest the harmful motifs in gluten molecules [25]. Gluten-blocking peptides, like the ones described in this work, could perhaps be one way to detoxify disease-inducing gluten peptides in the future.

By using a number of different panning conditions and gliadin proteins dissolved in either urea or NaHCO_3 _a large number of phage displaying different peptide sequences has been identified. To investigate if the identified peptides could interfere with the human anti-gliadin antibody and gliadin interaction, we mixed peptide-carrying phage with gliadin and pooled sera originating from patients with suspected CD. In that way we demonstrated that the phage indeed inhibited interactions between gliadin and human anti-gliadin antibody (Figure 4). For these analyses we used patient sera from a biobank. The sera were selected for having high titers of anti-gliadin antibodies (≤100 U/ml) and positive or high titers of anti-transglutaminase IgA antibodies, although we had no specific information regarding the patients' clinical diagnosis. The production of anti-gliadin antibodies is not specific to coeliac disease since slightly elevated serum concentrations are also found in other gastrointestinal disorders and even in normal individuals [51,52]. However, the levels of anti-gliadin antibodies in patients without coeliac disease seem to be much lower compared to our selected serum samples [53]. As our patients had high levels of both anti-gliadin and anti-transglutaminase antibodies it is likely that they had CD.

To verify whether the peptides, also when removed from the context of the phage, could interact with gliadin, we synthesised three peptides that were repeatedly identified in independent panning experiments and one control peptide that only interacted with BSA. We could then show that two of the peptides, P61 and P64 indeed interfered with the gliadin anti-gliadin antibody binding (Figure 6 and 7). In this case, two different monoclonal anti-gliadin antibodies provided by a commercial kit were used. In addition, by means of the biotin label attached to the peptides, we also showed in western blot experiments that P61 and P64 could bind to several of the separated and immobilised gliadin proteins (Figure 9).

Since we have so far only studied the nine gliadin-binding peptides that were most often picked up, we still have more than 150 additional peptides to test. Most likely, several of these peptides will also bind to gliadin. Since all individual peptides will bind to different sites on the gliadin complex, pooling of several different peptides could generate synergistic effects, and it should be possible to develop this concept in the direction of a drug against CD. However, many more experiments have to be performed, addressing issues like the stability of the peptide-gliadin interaction in chemical conditions likely to be encountered in the gut or in food preparation, the characterization of the actual binding sites in more detail, and the interaction with digestive enzymes and tissue transglutaminase etc. before any conclusions about the usefulness of these peptides in a therapeutic situation can be drawn.

## Conclusions

Finally, there are still several unanswered questions on the role of gliadin in the development of CD. Some of the gliadin-binding peptides presented here, labelled in different ways, could provide valuable tools for researchers in the field of CD to study localisation and uptake of various gliadin peptides in the small intestine.

## Methods

### Gliadin preparation

Gliadin was extracted from gluten (Sigma Aldrich, Stockholm, Sweden) as described [41] with some modifications. Essentially, 1.5 g gluten was dissolved in 20 ml 25 mM Na_2_SO_3_, vortexed 15 min at room temperature (RT) and centrifuged at 5000 *g *for 5 min. The pellet was washed in 20 ml 25 mM Na_2_SO_3 _and suspended in 70% ethanol by incubating at 70°C for 30 min with vortexing every 5 min. Undissolved material was eliminated by centrifugation, and the supernatant was incubated on ice for 2 h to precipitate the high molecular weight glutenin, which was eliminated by centrifugation for 10 min at 4°C. Subsequently the supernatant was mixed with 6 M NaCl in 70% ethanol to a final concentration of 256.67 mM NaCl and centrifuged for 10 min at 4°C. The supernatant that contained the gliadin-LMW-glutenin enriched fraction (in 70% ethanol and 256.67 mM NaCl) was stored at -80°C until further use. In addition, seeds (*ca *1 g) from *Surco *(wheat) and *Leon *(oat) varieties were ground and dissolved in 1 ml of 40% ethanol. The samples were centrifuged at 5000 *g *for 10 min and the supernatant stored at -20°C.

### Immobilisation of gliadin proteins

Gliadin (1-100 ng) prepared as above and diluted in 0.1 M NaHCO_3 _(pH 8.6) was incubated in 96-well microtiter (EIA/RIA) plates (Corning Inc. Corning, NY) at 4°C for 16 h. The amount of immobilized gliadin was quantified using the Anti-Gliadin IgG Kit (Biohit Oyi, Helsinki, Finland) according to the producer's protocol where the "positive control" patient serum provided in the kit was used as the primary anti-gliadin antibody, and labelled polyclonal anti-human IgG (goat) antibody was used as the secondary antibody. The positive signal was developed using the p-nitrophenyl phosphate solution (NPP) reagent and measured as absorbance at 405 nm.

### In vitro panning of phage display peptide library

The Ph.D. -12™ Phage Display Peptide Library kit, including *E. coli *ER 2738 host strain, was purchased from New England BioLabs (Beverly, MA). Selection of peptides was carried out according to the manufacturer's instructions. 25 μg of gliadin in 0.1 M sodium bicarbonate (pH 8.6) was coated onto 96-well microtiter plates (EIA plates) at 4°C overnight. Remaining surfaces in the wells were then blocked for 2 h at 4°C with 5% BSA diluted in 0.1 M sodium bicarbonate (pH 8.6) with 0.02% NaN_3_. Afterwards, approximately 1 × 10^11 ^plaque forming units (pfu) of phage were diluted in 100 μl of 1 × LIB (Low Ionic Strength buffer, 10 mM sodium phosphate, pH 6.0) with 0.5% BSA and 0.1% Tween-20, and incubated with gliadin for 1 h at RT with gentle shaking. The same procedure was used in negative control pannings but in this case the wells were just coated with BSA (no gliadin present). After phage incubation, the wells were washed ten times with LIB with 0.5% Tween-20. Unbound phage were discarded. Bound phage were eluted with 0.2 M glycine-HCl, 1% BSA (pH 2.2) and amplified by infecting *E. coli *ER2738 host cells. After 4.5 h of growth at 37°C phage were removed from bacterial cells by centrifugation. The phage present in the supernatant were precipitated by adding 1/6 volume of PEG/NaCl solution (20% w/v polyethylene glycol-8000; 2.5 M NaCl), and incubated for 16 h at 4°C. The precipitate was resuspended in a small volume of LIB, and amplified elutes were titrated to determine phage concentration. Typically, the panning procedure was repeated five times after which phage were plated and random plaques were picked. After amplification, phage were purified by precipitation in PEG/NaCl followed by resuspension in 1/50 volume of the original volume in 1 × LIB with 0.02% NaN_3 _and stored in aliquots at 4°C. These phage were then used in the binding specificity and affinity experiments and for DNA extraction.

### DNA sequencing

Single-stranded phage DNA was isolated by incubation in iodide buffer (4 M NaI, 1 mM EDTA in 10 mM Tris-HCl, pH 8.0) to denature the phage coat protein. Released DNA was then precipitated in 70% ethanol. Purified DNA was sequenced by Microgen Inc. (Seoul, Korea) and MWG Biotech AG (Martinsried, Germany).

### Phage recovery experiment

Coated and blocked (as described above) microtiter plate wells were washed three times with 0.1% LIBT (LIB buffer with 0.1% Tween-20). Selected phage were serially diluted in 0.1% LIBT buffer and 100 μl was added to the wells. After addition of 1% BSA the wells were incubated for 1 h at 37°C. Control wells were incubated in the same buffer but without phage. Next, the wells were washed six times with 0.5% LIBT to remove the unbound phage. The remaining phage were eluted with glycine-HCl (pH 2.2) in 1% BSA and phage titers were determined.

### Patient antisera

The serum samples were obtained from a biobank at the immunological laboratory, Sahlgrenska University hospital, Gothenburg, Sweden. The samples were selected for high titers (≤100 U/ml) of anti-gliadin IgA antibodies and for positive or high titers of anti-transglutaminase IgA antibodies. The serum samples were prepared according to standard procedure, i.e. blood was drawn into unprepped tubes and serum was collected by centrifugation at 3000 *g*. Serum was diluted (1:500) with dilute buffer (same as dilute buffer from Anti-Gliadin IgG kit). The serum used here was a pool from 20 different anonymous patients.

### Phage ELISA

Since the phage display was done on a mixture of different gliadin proteins, potentially a lot of different peptides could bind to the coated proteins. To block out as many peptides as possible in the same experiment pooled polyclonal patient sera isolated from 20 different patients with suspected CD were used.

Microtiter plate wells were coated with 100 μl of gliadin proteins (0-100 μg/ml) dissolved in 0.1 M NaHCO_3 _(pH 8.6) and incubated overnight at 4°C. Subsequently, the wells were blocked with 200 μl of blocking buffer (5% BSA in 0.1 M NaHCO_3_, pH 8.6; with 0.02% NaN_3_) for 2 h at 4°C and washed three times with 0.1% LIBT (LIB with 0.1% Tween-20). Phage (1 × 10^11^) carrying different peptide sequences in 100 μl blocking buffer were transferred to the coated wells and incubated at 37°C for 1 h. Unbound phage were removed by washing six times with 0.5% LIBT (1 × LIB buffer with 0.5% Tween-20). After this, 100 μl pooled patient antiserum (diluted 1:500 with dilution buffer from the Anti-Gliadin IgG kit, Biohit Oyi, Helsinki, Finland) was added. After the 30 min incubation at RT the wells were washed four times with 1 × ELISA washing buffer (Phosphate Buffered Saline, pH 7.2, 0.05% Tween-20, Biolegend, San Diego, CA). For detection, 100 μl of AP-linked, goat anti-human IgG (Invitro/Biolabs, Beverly, MA) diluted (1:4500) with dilution buffer from the Anti-Gliadin IgG kit was added to the wells and incubated for 30 min at RT. After washing four times with 1 × ELISA washing buffer, 100 μl of Nitrophenyl Phosphate Disodium substrate solution (NPP) (Invitrogen, Madison, WI) was added and incubated for 30 min at RT. Finally, the signal was detected by measuring absorbance at 405 nm in a microplate reader.

### Peptide synthesis

Four peptides, denoted P61, P64, P22 and CP31 were synthesized at >95% purity by Bio-Synthesis Inc. (Lewisville, TX), with biotin added to the N terminus. Peptides were dissolved in 150 μl DMF (dimethylformamide) and diluted to 1 ml with 0.05 M phosphate buffer containing 0.15 M NaCl, pH 7.4 (Peptide Dilution Buffer, PDB) to a final concentration of 16.7 μM. Aliquots were stored at -20°C until further use.

### Binding of synthetic peptides to gliadin proteins

100 μl, corresponding to 1.67 nmoles (10^15 ^molecules) of the synthesized peptides were added to gliadin-coated microtiter wells (1-100 ng) and was incubated in 0.1 M NaHCO_3 _(pH 8.6) for 1 h at 37C.

The wells were washed four times with 1 × ELISA buffer (diluted from 20 x ELISA washing buffer, Biolegend, San Diego, CA) after which 100 μl anti-biotin AP-linked antibody (http://www.cellsignal.com) diluted 1:3000 was added. After the 30 min incubation at RT, and washing (four times) with 1 × ELISA buffer, 200 μl of 1 × NPP substrate (Invitrogen, Carlsbad, CA) was added to the wells. After the 30 min incubation at RT in the dark 100 μl of stop solution was added, the plates were shaken, and the signal was read at 405 nm.

### Inhibition of gliadin-anti-gliadin antibody binding by selected synthetic peptides

Gliadin was prepared as described and diluted to a final concentration of 100, 50, 30, 10, 5, 3, 1 and 0.5 ng/ml in 200 μl dilution buffer provided in the Immunotech ELISA kit (Radiová 1, Prague, Czech Republic). Each gliadin dilution was incubated with 1.67 nmoles (10^15 ^molecules) of the synthetic peptides P64, P61, P22, CP31 and a control with only 0.1 M NaHCO_3 _buffer at RT for 1 h. The gliadin/peptide mixtures were then added to microtiter wells coated with two different anti-gliadin monoclonal antibodies provided with the Immunotech Gliadin ELISA kit. As internal calibrators, 0 and 9 ng gliadin solutions were added to separate wells. Wells with the different mixes were incubated for another hour at RT. Afterwards bound gliadins were quantified using a polyclonal antibody (horseradish peroxidase conjugate) that binds to the solid phase antibody-antigen complex. Bound secondary antibody was quantified using TMB substrate (tetramethylbenzidine) as described in the kit. Positive signals were given as absorbance units at 450 nm.

### Concentration dependency of peptide gliadin-anti-gliadin antibody binding inhibition

Gliadin was diluted to a final concentration of 1 ng/ml in 200 μl 0.1 M NaHCO_3 _dilution buffer as described and mixed with 1.67 nmoles, 0.167 pmoles, and 0.0167 fmoles (10^15^, 10^9^, 10^5 ^molecules) respectively of the biotin-labelled synthetic peptides P61, P64, and CP31, dissolved in 0.1 M NaHCO_3 _buffer. After incubation for 1 h at RT the gliadin/peptide mixtures were then added to the anti-gliadin antibody coated microtiter wells and incubated for another hour. Secondary antibody was thereafter added and quantified as absorbance units at 450 nm as described above.

### Dot blot assay

4 μl of serial dilutions of gliadin proteins in 0.1 M NaHCO_3 _(pH 8.6) were spotted onto 0.45 μm nitrocellulose membranes, air dried, and subsequently quenched by soaking into 5% non-fat milk in PBS overnight at 4°C. Blocking solution was removed by washing the membranes with PBST (137.9 mM NaCl, 1.47 mM KH_2_PO_4_, 8.1 mM Na_2_HPO_4_, 2.68 mM KCl, 0.05% Tween-20, pH 7.4). Biotinylated blocking peptides were diluted in PBS and incubated with the membranes for 3 h at RT with gentle shaking. After subsequent washing with PBST (four times), anti-biotin AP-linked antibody (diluted 1:3000 with dilute buffer, 50 mM PBS, pH 7.2, 0.05% Tween-20) was incubated with the membranes for 2 h with gentle shaking. Finally, the membranes were washed four times with PBST-0.05%, once with PBS and the AP-substrate was added. The images were developed with immune-star™ AP chemiluminescent protein detection system (Bio-Rad Laboratories, Sundbyberg, Sweden).

### Western blot analysis

Gliadin proteins were separated during SDS-PAGE on 12% Tris-glycine gels. The separated proteins were transferred in transfer buffer (48 mM Tris, 38.6 mM glycine, 1.6 mM SDS, 20% methanol) to nitrocellulose membranes (Amersham/Biosciences, Sweden) for 2.5 h at 90 mA in a semi-dry electroblotting unit (Z34050-2, Sigma, Stockholm, Sweden). After the protein transfer the membranes were washed with washing buffer (PBS, 0.05% Tween-20) and blocked with 3% BSA in PBS overnight at 4°C. For development of the biotin signal, the same protocol as in the Dot blot assay was used.

The study was approved by the Human Research Ethics Committee of the Medical Faculty, Gothenburg University, Gothenburg, Sweden with the permission number 144-06. The serum samples were obtained from a biobank at the immunological laboratory, Sahlgrenska University hospital, Gothenburg. Biobank samples were selected for high titers of gliadin-specific IgA antibodies and for positive or high titers of anti-tTG antibodies. According to the Swedish biobank law, the serum samples were completely impersonalized, which means that the samples cannot be linked to any patient, his or her personal data, or to the clinical evaluation.

## Authors' contributions

TC developed and optimised the phage display technology and performed most of the experiments to test the peptides. KH participated in the gluten purification and gave suggestions during method development. SÖ provided the human antibodies and helped with ELISA experiments. OO and ASS planned the project. OO supervised the work and wrote the manuscript together with TC and KH. All authors read and approved the final manuscript.
